# Host Response Biomarkers for Sepsis in the Emergency Room

**DOI:** 10.1186/s13054-023-04367-z

**Published:** 2023-03-21

**Authors:** Oren Turgman, Michiel Schinkel, Willem Joost Wiersinga

**Affiliations:** 1 Center for Experimental and Molecular Medicine, Amsterdam Institute for Infection and Immunity, Amsterdam UMC, Location Academic Medical Center, University of Amsterdam, Amsterdam, The Netherlands; 2 Division of Infectious Diseases, Department of Medicine, Amsterdam UMC, Location Academic Medical Center, University of Amsterdam, Amsterdam, The Netherlands

## Abstract

This article is one of ten reviews selected from the Annual Update in Intensive Care and Emergency Medicine 2023. Other selected articles can be found online at https://www.biomedcentral.com/collections/annualupdate2023. Further information about the Annual Update in Intensive Care and Emergency Medicine is available from https://link.springer.com/bookseries/8901.

## Introduction

Sepsis is a medical emergency currently defined as life-threatening organ dysfunction caused by a dysregulated host response to infection [1]. With a recent estimate 6 of 11 million sepsis-related deaths out of 48.9 million yearly sepsis cases, it is a 7 global health priority [2]. Current sepsis treatment guidelines recommend general 8 measures, such as antibiotic treatment, source control, and resuscitation [3]. The 9 heterogeneity of the sepsis syndrome however makes early and consistent diagnosis difficult [4] and has resulted in a lack of sepsis-specific treatments [5, 6]. An essential factor limiting our ability to detect sepsis is the lack of clinically relevant bio- markers for the early phases of the syndrome.

The benefits of early diagnosis and treatment have been well-studied and protocolized in specialties such as trauma medicine, cardiology (e.g., myocardial infarction and cardiac arrest) and neurology (e.g., stroke management), but less so in the field of sepsis [7]. This can potentially lead to longer time-to-antibiotics and higher mortality [8]. Sepsis patients often undergo their first extensive evaluation in the emergency room (ER). Decisions made at this stage, such as choice of antibiotic treatment and discharge destination, are likely to highly impact the rest of the hospital stay. Biomarkers are able to reduce the heterogeneity among sepsis patients in the ER and could improve their care.

**Fig. 1:**
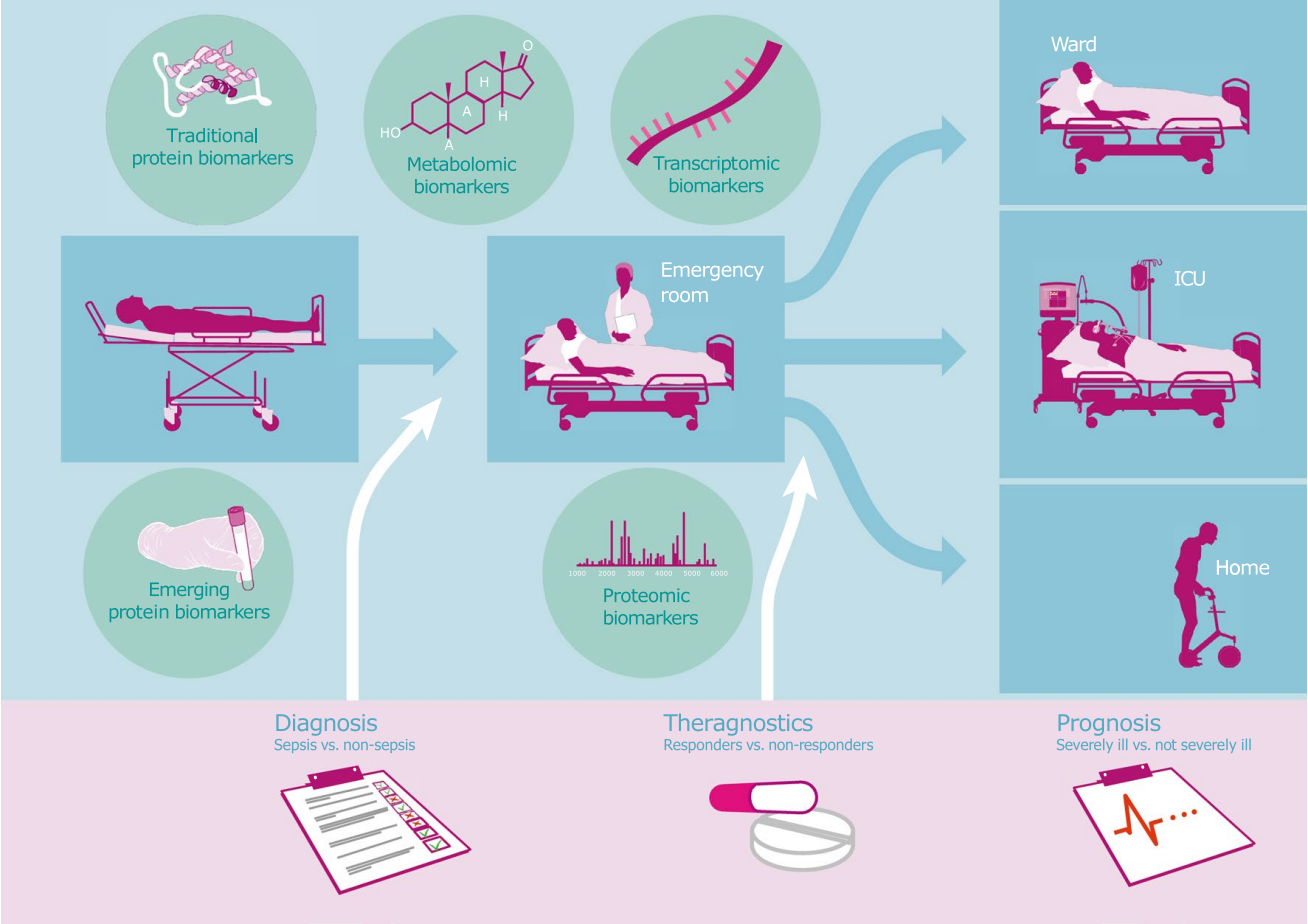
Host response biomarkers for sepsis in the emergency room (ER). A visualization of the patient flow through the ER and of the different types of sepsis biomarkers and their respective roles in this process

This review aims to provide a high-level overview of sepsis biomarkers that are relevant for the ER setting. It focuses on markers found in the systemic compartment and includes traditional and emerging protein biomarkers, but also those arising from the fields of transcriptomics, proteomics, and metabolomics (Fig. 1).

## Biomarkers in the Context of Sepsis

Biomarkers can be defined as any objective, reproducible characteristics by which (patho)physiologic processes can be identified and measured [9]. Within the field of sepsis, one can differentiate between diagnostic, prognostic, and therapeutic bio- markers [10, 11]. Diagnostic biomarkers differentiate between infectious and non-infectious disease or help identify specific pathogens. Prognostic biomarkers are useful for assessing the risk of poor outcomes in septic patients and can help us stratify patients by their risk profiles. Lastly, therapeutic biomarkers are used to assess the effectiveness of a treatment. In recent years, biomarkers have been used for a fourth category: theragnostics. Theragnostics describes an approach in which patients are stratified based on specific biomarkers or pathways that might be more susceptible to specific intervention [5, 12]. Also termed predictive enrichment, this approach stands in contrast to prognostic enrichment wherein patients with a higher likelihood of having a certain outcome are identified. Prognostic enrichment in trials can improve power and reduce the number of patients needed [12]. This is underscored by recent studies in patients with severe coronavirus disease 2019 (COVID-19) in which C-reactive protein (CRP) and soluble urokinase plasminogen receptor (suPAR) plasma levels were successfully used to guide treatment with respectively anti-interleukin (IL)-6 and anti-IL-1 [13, 14]. In the later study, allocation of patients to treatment groups was guided by suPAR, a biomarker that predicts progression to severe respiratory failure or death in COVID-19 patients. The study only included patients with suPAR levels >6 ng/ml, and randomized them to receive either IL-1 receptor antagonist or placebo in addition to the standard-of-care treatment. The sepsis field is moving from traditional ways of diagnosis and treatment towards a precision medicine approach, in which more homogeneous patient groups with shared pathophysiologic pathways are identified that are amenable to a specific treatment [15]. Biomarkers will be essential to expedite this transition.

## Traditional Biomarkers: CRP and Procalcitonin (PCT)

From the hundreds of biomarkers evaluated for diagnosing infection and sepsis, only a handful are used by clinicians on a large scale. Although non-specific for the diagnosis of sepsis, CRP and procalcitonin (PCT) are often used to detect inflammaion because of their high sensitivity [16]. CRP is an acute-phase reactant protein synthesized by the liver, primarily induced by IL-6 [17], whereas PCT is a precursor for the calcitonin hormone, normally made in the thyroid gland. When compared to CRP, PCT levels increase faster after stimulation, reach their peak faster, and also decline faster after resolution of infection [18]. These are desirable characteristics for a biomarker, especially in the ER, as they describe the current state of a patient more accurately. A systematic review, which assessed the accuracy and clinical value of PCT for diagnosis of sepsis in intensive care unit (ICU) and ER patients, reported an area under the curve (AUC) value of 0.85 (95% confidence interval (CI) 0.81–0.88) for diagnosis of sepsis [19]. One of the included studies, among ER patients with suspected infection, reported an AUC value for PCT of 0.79 for diagnosis of sepsis, which was better than that of IL-6 (AUC 0.70) or CRP (AUC 0.67) [20]. This finding was confirmed in a recent systematic review that specifically addressed the role of PCT in the ER setting [21]; according to most included studies, PCT performed better than CRP or lactate as a diagnostic biomarker for sepsis. As a predictor of adverse outcomes, the data on PCT are more inconsistent. In some studies PCT was superior to CRP and lactate, but in others it was not [21]. There is, however, solid evidence that PCT can be of added value for effectively and safely reducing the duration of antibiotic therapy in critically ill patients. This is underlined by a recent meta-analysis that included studies in patients with sepsis and/or respiratory infection, demonstrating that duration of antibiotic treatment was significantly reduced in both groups when PCT was used as a guiding biomarker compared with clinical evaluation alone [18]; as a secondary outcome, in-hospital mortality was also significantly lower among these patients [18]. Currently, the Surviving Sepsis Campaign (SSC) guidelines advise against using PCT to aid the decision of when to start antimicrobials and suggest clinical evaluation alone [3].

However, PCT use is suggested to help decide when to discontinue antimicrobials among adult patients with sepsis where the optimal duration of therapy is unclear [3].

## Emerging Biomarkers

Over 250 biomarkers for sepsis have been evaluated in clinical and experimental studies [10]. Examples of emerging biomarkers that have been studied in the ER setting for either diagnostic or prognostic value include presepsin, IL-6, lipopolysaccharide-binding protein (LBP), pancreatic stone protein (PSP), bactericidal/permeability-increasing protein (BPI), group II phospholipase A2 (PLA2GIIA), brain natriuretic peptide (BNP), soluble triggering receptor expressed on myeloid cells-1 (sTREM-1), suPAR, pro-adrenomedullin (MR-proADM), macrophage migration inhibitory factor (MIF), heparin-binding protein, D-dimer, soluble IL-2 receptor alpha chain (sCD25), and cell-free DNA [10]. Many more potential sepsis biomarkers have been studied in non-ER patient groups, but these are outside the scope of this review. Those discussed herein were selected based on the presence of recent literature, the number of included patients in the relevant studies, and the setting in which the biomarker was tested (i.e., in the ER). A full overview of sepsis biomarkers can be found elsewhere [10, 11].

## Presepsin

Presepsin, first described as soluble CD14 subtype in 2005 [22], is a soluble fragment of CD14, a broad-spectrum affinity component of the innate immune system. Serum presepsin levels start to rise 2 h after infection and already reach their peak after 3 h, making it a potentially valuable early biomarker for sepsis in the ER [23, 24]. A 2013 study from China in 859 ER patients with at least two diagnostic criteria for systemic inflammatory response syndrome (SIRS), showed superior performance characteristics of presepsin compared to PCT for sepsis diagnosis (AUC of 0.820 vs. 0.724 for PCT) as well as prediction of severe sepsis (AUC 0.840 vs. 0.741) [25]. A more recent meta-analysis from 2017 concluded that presepsin had a pooled sensitivity of 0.84 (95% CI 0.80–0.87) and specificity of 0.76 (95% CI 0.67–0.84) for diagnosing sepsis among critically ill patients (admitted to the ER, ICU or cardiac care unit) [23]. Interestingly, when subgroups were analyzed, presepsin had better specificity in ER patients when compared to ICU patients (0.82, 95% CI 0.69–0.91 vs. 0.64, 95% CI 0.51–0.76) with an AUC indicative of higher accuracy in the ER group (0.91 vs. 0.85) [23]. However, when compared to CRP or PCT in ER patients, presepsin did not perform better in terms of diagnosing sepsis in this meta-analysis [23]. Finally, in a recent South Korean prospective observational study among 420 ER patients, presepsin performed better than CRP in terms of differentiating between non-infectious organ failure, sepsis, and septic shock, defined according to Sepsis-3 criteria [24]. However, presepsin was described as having equal performance compared to PCT [24]. In this study, patients with high serum presepsin levels (≥ 821 pg/ml) had a higher mortality rate compared to patients with lower levels (33.3% vs. 18.4% respectively) [24]. These results indicate that presepsin has value as a diagnostic and prognostic biomarker for sepsis in the ER, but whether it outperforms PCT or CRP has not been conclusively shown.

## sTREM-1

TREM-1 is a member of the immunoglobulin superfamily expressed on neutrophils and monocytes [26]. During bacterial and fungal infections, TREM-1 is upregulated, which causes release of sTREM-1. In a 2021 meta-analysis, sTREM-1 was described as having a sensitivity of 0.85 (95% CI 0.76–0.91) and a specificity of 0.79 (95% CI 0.70–0.86) for differentiating sepsis from SIRS among critically ill patients from various countries worldwide [27]. sTREM-1 was also able to predict 28-day mortality with a sensitivity of 0.80 (95% CI 0.66–0.89) and a specificity of 0.75 95% (CI 0.70–0.86) [27]. Looking at the ER specifically, a recent Taiwanese study investigated the prognostic value of sTREM-1 in ER patients with sepsis [28]. Significantly higher and sustained levels of sTREM-1 were found among non-survivors on days 1, 4, and 7 after admission. sTREM-1 and sequential organ failure assessment (SOFA) scores were the only factors independently associated with sepsis-related death, with AUC values of 0.726 (95% CI 0.613–0.838; p = 0.028) and 0.705 (95% CI 0.602–0.808; p = 0.042), respectively [28]. Taken together, sTREM-1 has been shown to have diagnostic and prognostic value as a biomarker for sepsis in critically ill patients. However, studies that evaluate the added value of sTREM-1 on meaningful patient-related outcome parameters in comparison to other biomarkers specifically in the ER are lacking.

## Proadrenomedullin

MR-proADM, derived from adrenomedullin (ADM), has been shown to play a role in preserving the integrity and stability of the endothelium after severe infection [29]. ADM acts directly on the sympathetic nervous system, causing arterial and venousvasodilation, natriuresis, bronchodilation, and positive inotropy. MR-proADM as a compound is more stable than ADM and accurately reflects ADM levels [30]. A recent systematic review that investigated MR-proADM as an early biomarker for sepsis, included 11 studies with 1176 sepsis patients and 823 controls [30]. ER, ICU, and ward patients were included. Diagnostic criteria for sepsis varied between studies. Pooled data concerning the ability of MR-proADM to diagnose sepsis showed a sensitivity of 0.83% (95% CI 0.79–0.87%) and specificity of 0.90% (95% CI 0.83–0.94%). Less is known about MR-proADM as a prognostic biomarker of sepsis. MR-proADM levels measured within 24 h of ICU admission have been shown to be positively correlated with organ failure in patients with sepsis [30]. Among ER patients, MR-proADM was the only biomarker associated with blood culture status, i.e., low levels were correlated with a negative blood culture, when compared to CRP, PCT, lactate, soluble PLA2GIIA, sTREM-1, presepsin, and sCD25 [31]. An English study among ER patients with COVID-19, found that MR-proADM could predict 30-day mortality more accurately than CRP, PCT, white blood cell count, and lymphocyte or neutrophil count. Elevated MR-proADM levels were also associated with critical care admission and non-invasive ventilation among these patients [29]. Taken together, MR-proADM can assist in the diagnosis of sepsis as well as prediction of severe disease among patients with suspected infection in the ER. However, there are no studies addressing its true potential for improving the outcome of patients in the ER with (suspected) sepsis.

## Transcriptomics

Recent advances in omic technologies have shifted the focus from traditional protein biomarkers to the fields of genomics, transcriptomics, proteomics, and metabolomics [32]. Transcriptomics and proteomics have been studied the most in the context of sepsis biomarkers. By analyzing relevant data on a molecular level, it has become possible to identify sepsis and stratify patients according to information pertaining to cellular proteins, metabolites, genes, and their expression. This allows for more homogenous cohorts of patients that share biological similarities, which might open the door for effective treatment strategies, designed to address a certain pathophysiological pathway.

A recent study aimed to identify novel transcriptional diagnostic and risk stratification biomarkers among ER and ICU patients from various countries worldwide with suspected infection and at least two SIRS/Sepsis-1 criteria [33]. Using unsupervised machine learning, several immune-related processes were found to differ among severely ill patients (24-h SOFA score >5) compared to patients who were less sick. For example, neutrophil degranulation was the most enriched pathway among severely ill patients, indicating that sepsis severity in the early stages is strongly associated with neutrophil activity. Interferon-gamma (IFNγ) was notably downregulated among non-survivors. Using RNAseq data, a 40-gene classification set was identified that was able to categorize patients into one of five mechanistic endotypes with an accuracy of 96% [33]. Different endotypes were found to have different enriched pathways and severity of disease. For example, neutrophilic-suppressive (NPS) and inflammatory (INF) endotypes were associated with more severe disease based on organ failure probability, SOFA scores, length of hospital stay, requirement for oxygen supplementation, and positive blood culture tests. The NPS endotype was found to include the most immunosuppressed patients with severe disease, with downregulation of IFN signaling, CD28 co-stimulation, and programmed-death (PD)-1 signaling. Conversely, the INF endotype, which also included the most severely ill patients, was found to have upregulation of multiple inflammatory pathways, such as IFNγ and TNF-α signaling [33].

In a different study from the USA, using 29 preselected mRNA inputs inflammatix-bacterial-viral-non-infected-version 1 (IMX-BVN-1) was created, a neural network classifier trained to differentiate between bacterial, viral, and non-infectious disease among ICU patients [34]. When infection adjudications were unanimous, IMX-BVN-1 was shown to have AUC values of 0.86 (bacterial-vs.-other), 0.85 (viral-vs.-other), and 0.82 (non-infected-vs.-other). In a later validation study among 312 ER patients with suspected acute infections and at least one abnormal vital sign, IMX-BVN-2 (an updated version of IMX-BVN-1) was described to have AUC values of 0.82–0.90 for differentiating between bacterial infection and no infection [35]. This was superior to PCT (AUC 0.80–0.89), CRP (0.79–0.84), and white blood cell count (0.69–0.77). AUC values ranged due to varying degrees of certainty in the retrospective adjudication of infection status. When differentiating between viral infections and no infection, IMX-BVN-2 showed an AUC value of 0.82–0.83, whereas PCT, CRP, and white blood cell count could not differentiate between these groups (AUC <0.4). Most recently, IMX-SEV-2, a variant of IMXBVN-2 developed for predicting disease severity in patients with sepsis, was described to have an AUC value of 0.84 (95% CI 0.76–0.93) for predicting in hospital mortality [36]. This was superior to lactate (AUC 0.76; 95% CI 0.64–0.87), qSOFA (0.68; 95% CI 0.57–0.79), and the National Early Warning Score 2 (0.75; 95% CI 0.65–0.85) (p = 0.015, 0.001, and 0.013, respectively).

Other tools that use transcriptomic data and have been developed as biomarkers for sepsis are SeptiCyte Lab [37], the sepsis mortality score (SMS) for prediction of death in septic patients [38], and the FAIM3:PLAC8 ratio, originally derived to identify community-acquired pneumonia in ICU patients but later studied as a bio-marker for sepsis [39]. These promising tools await validation in an ER setting where clinical sepsis is not always overt.

## Proteomics and Metabolomics

The proteome is defined as the entire set of proteins that can be expressed by a genome while the metabolome is defined as all the small molecules in cells that are a product of the genome and of genome-environment interactions [40]. These novel analytes have been used to diagnose sepsis, reveal profiles of patients that relate to clinical outcomes, and identify future targets for intervention. Techniques such as chromatography and mass-spectrometry have enabled us to study very small particles that provide information of underlying biological processes. A novel technique, Raman spectroscopy, uses light scattering to non-destructively provide a structural fingerprint by which particles can be identified and has been used for identifying pathogens and metabolites, such as IL-6 [41, 42]. In a cohort of 63 critically ill patients with sepsis and 43 controls, Chen et al. used a combined proteomics and metabolomics approach to identify specific profiles in amino acid metabolism that could differentiate between patients with sepsis and healthy controls, with AUC values ranging from 0.81 to 0.96 [43]. In a validation group, the AUC value ranged from 0.62 to 1.00. Among the most discriminative metabolites were fatty acids and glycerophospholipids, which have roles in energy production and lipid signal transduction, respectively. Pathways and proteins involved in the acute inflammatory response, Toll-like receptor (TLR) signaling, defense response, and activation of myeloid cells were correlated with sepsis and sepsis-associated kidney injury.

Multi-omics analysis has also been used to develop tools for triage of pediatric sepsis. A study published in 2015 used metabolomic and proteomic profiling data to assess the accuracy of these profiles in retrospectively differentiating between pediatric patients with sepsis who were admitted to the pediatric ICU (PICU) (n = 94) and those who presented to the ER but were not admitted to the PICU (n = 81).

Reported AUC values ranged from 0.88–0.96 [44]. Multiomic-based profiles that can be used to predict severity of disease and necessity for certain treatments are valuable, especially when certain expertise is not available. In a more recent study, van Houten et al. investigated whether a host-protein-based assay could differentiate between bacterial and viral infection among preschool children [45]. The assay integrates concentrations of three biomarkers: TNF-related apoptosis-inducing ligand (TRAIL), IFNγ induced protein-10 (IP10), and CRP. Patients (n = 577) were aged 2–60 months and were included from four hospitals in the Netherlands and two hospitals in Israel. Patients were either diagnosed with lower respiratory tract infection or had a clinical presentation with fever with unknown source. Reference diagnoses were adjudicated by a panel of three experienced pediatricians, based on all clinical information including follow-up at 28 days. AUC values for differentiating between viral and bacterial infection ranged from 0.90–0.92, depending on the degree of certainty of the adjudicating panelists [45].

A metabolomics approach has also been used to diagnose bacteremic sepsis among Swedish adult ER patients [46]. Of these patients, 65 had laboratory-confirmed bacteremia and fulfilled the 1992 criteria for sepsis (i.e., SIRS criteria plus infection). The remaining 45 patients were initially also suspected of bacteremic sepsis, but were found to have negative blood cultures and laboratory-confirmed alternative diagnoses. After analysis, six metabolites were integrated into a diagnostic tool, which was tested in a subgroup of the cohort and was found to be able to predict bacteremic sepsis with an accuracy of 88.1% [46]. Metabolomics has also brought possibilities for personalizing drug treatment as was exemplified in a study in the USA in 21 patients with vasopressor-dependent septic shock treated with l-carnitine [47]. Responders to l-carnitine had significant changes in their metabolomic signatures. Taken together, these data show that metabolic signatures can be used for the diagnosis of sepsis, for the stratification of patients towards a specific treatment, and as a marker of disease severity.

## Conclusion

Biomarkers for sepsis in the ER can be of diagnostic, prognostic, and therapeutic value. Theragnostics entails the use of biomarkers for predictive or prognostic enrichment [11]. A large number of sepsis markers has been identified and studied recently, but only a few are being used widely in everyday clinical practice [10]. Ideally, a biomarker can diagnose sepsis, predict its severity, and help to ascertain appropriate treatment. It should be able to stratify patients according to their disease severity or susceptibility to a specific treatment. Unfortunately, biomarkers that can achieve these goals consistently in the heterogeneous population found in the ER setting have not yet been identified. Widely known and used are CRP and PCT, which can have important roles in the ER but do not meet the aforementioned criteria for the ideal biomarker. Emerging protein biomarkers, such as presepsin and MR-proADM, have shown promise in several specific homogeneous populations but validation in large and diverse cohorts is lacking. From the field of omics, several promising new techniques have emerged, enabling health care providers to integrate high-dimensional data, which can increase power and efficiency for identifying clinically relevant biomarkers for sepsis in the ER setting [32]. However, further validation of these biomarkers should be done first. Furthermore, their effect on clinically meaningful patient-related outcome measures, let alone their cost-effectiveness, is often ill-defined and should be the subject of further studies. It is very unlikely that a single biomarker will provide us with the answers to all our needs. Panels of biomarkers have been shown to be more effective in many aspects than single ones [48]. The field of biomarkers for sepsis in the ER is developing rapidly. In the near future, management of sepsis in the ER is likely to be guided by repeated measurements of biomarker arrays, which utilize point-of-care tests. These tests limit hands-on time and reflect abnormalities in host response pathways that are amenable to targeted therapeutics.

## Data Availability

Not applicable.
